# Household income modifies the association of insurance and dental visiting

**DOI:** 10.1186/1472-6963-13-432

**Published:** 2013-10-23

**Authors:** Olga Anikeeva, David S Brennan, Dana N Teusner

**Affiliations:** 1 Flinders University Disaster Research Centre, School of Nursing and Midwifery, Flinders University, Adelaide, SA 5001, Australia; 2 Australian Research Centre for Population Oral Health (ARCPOH), School of Dentistry, The University of Adelaide, Adelaide, SA 5005, Australia

**Keywords:** Access, Dental visiting, Income, Insurance

## Abstract

**Background:**

Dental insurance and income are positively associated with regular dental visiting. Higher income earners face fewer financial barriers to dental care, while dental insurance provides partial reimbursement. The aim was to explore whether household income has an effect on the relationship between insurance and visiting.

**Methods:**

A random sample of adults aged 30–61 years living in Australia was drawn from the Electoral Roll. Data were collected by mailed survey in 2009–10, including age, sex, dental insurance status and household income.

**Results:**

Responses were collected from n = 1,096 persons (response rate = 39.1%). Dental insurance was positively associated with regular visiting (adjusted prevalence ratio (PR) = 1.18; 95% CI: 1.01-1.36). Individuals in the lowest income tertile had a lower prevalence of regular visiting than those in the highest income group (PR = 0.78; 95% CI: 0.65-0.93). Visiting for a check-up was less prevalent among lower income earners (PR = 0.65; 95% CI: 0.50-0.83). Significant interaction terms indicated that the associations between insurance and visiting varied across income tertiles showing that income modified the effect.

**Conclusions:**

Household income modified the relationships between insurance and regular visiting and visiting for a check-up, with dental insurance having a greater impact on visiting among lower income groups.

## Background

In Australia, the majority of adults are ineligible to access publicly provided dental care and must pay for dental services, either by making the payments directly or by purchasing dental insurance, which provides partial reimbursement. Public provision of dental care is highly rationed, via waiting lists and triaging systems; therefore, even amongst those who are eligible for publicly funded care, a substantial proportion access care in the private sector [1,2].

Dental insurance has been found to be associated with higher rates of visiting for a check-up and regular dental visiting, [3-11] and was correlated with patient acceptance of prescribed dental treatment, [12] which suggests that insured individuals may face fewer financial barriers to comprehensive dental care. In addition, it has been found that while most individuals obtain basic dental care regardless of their insurance status, dental insurance is associated with use of more expensive dental services, further indicating the capacity of insurance to reduce financial barriers [13].

Income is associated with dental visiting and dental health outcomes, with socioeconomically disadvantaged adults more likely to report a lower frequency of dental visiting, a higher number of missing teeth and poorer self-rated oral health [1,4,6,7,10,11,14]. Lower income individuals were found to have higher odds of visiting a dentist for the purpose of pain relief and were more likely to receive extractions than their higher income counterparts [3,4,15-17]. This may reflect the ability of higher income earners to pay for comprehensive dental care, while those with low incomes may delay visiting a dentist until they experience dental problems [4]. Studies have suggested that these associations could not be attributed to personal neglect, such as poor self-care practices or negative attitudes towards dental visiting, among low income individuals [14]. In fact, adults from lower socioeconomic backgrounds were equally inclined to practice dental self-care as their higher socioeconomic status counterparts. Therefore, distal factors, such as financial barriers to accessing care or long waiting periods for public dental services, may be contributing to the observed associations between income, dental visiting and poor oral health [14,17].

The aim of this study was to investigate whether household income modifies the relationship between dental insurance and dental visiting. While positive associations between dental visiting and insurance have been explored, little Australian research has investigated whether these relationships are consistent across income groups. Understanding variations in associations is important to oral health policy that may be targeted to specific income groups.

## Methods

A random sample of 3000 adults aged 30–61 years living in Australia was drawn from the Electoral Roll by the Australian Electoral Commission. Sample size was determined by using estimates of percentage of persons making a dental visit in the last year (reflecting access to care) and percentage of persons receiving extractions (for comprehensiveness of care). Calculations were made based on comparisons of proportions using an alpha level of 0.05 and a beta of 0.80. The largest required sample size was n = 336 per group for comprehensiveness of care, which, allowing for 3 levels of disaggregation, would require a total of 1,008 subjects.

Data were collected by mailed self-complete questionnaires in 2009–2010, with four follow-up mailings to non-respondents at intervals of approximately three to four weeks. Questionnaires collected demographic characteristics, household income, private health insurance details, dental visiting patterns, oral health status and dental behaviours.

### Outcome variables

The outcome variables were regular dental visits (based on the question 'On average how often do you visit a dental professional?’) and last visited for a check-up (based on the question 'What was the main reason for your last dental visit?’). The index category for regular visits were those who on average made a dental visit at least once every two years and the index category for reason for visit were those whose last dental visit was for a check-up.

### Explanatory variables

The main explanatory variables were dental insurance status and household income. Dental insurance was coded as insured or uninsured. Household income per year was collected in seven categories (up to $20,000, $20,001 to $40,000, $40,001 to $60,000, $60,001 to $80,000, $80,001 to $100,000, $100,001 to $120,000 and more than $120,000) and coded into approximate tertiles.

Other explanatory variables comprised sex, age and tooth brushing. Age was determined by collecting year of birth information from respondents and coded into age groups of 30–39, 40–49 and 50–61 years. Tooth brushing was included as a proxy measure for individual orientation towards personal oral health care and was coded as those who brushed twice a day or more or those who brushed less than twice a day.

### Analysis

Data were analysed using SAS version 9.3. The analyses were restricted to dentate persons. The representativeness of the sample was assessed by comparing a selection of sociodemographic, socioeconomic, dental behaviour and oral health characteristics with population estimates. Unadjusted associations between the dental visiting outcome variables and the main explanatory variables and other variables were examined (Chi-square test, p < 0.05). In addition, the associations between insurance status and the dental visiting outcome variables, stratified by household income, were calculated. Prevalence ratios adjusted for explanatory variables were estimated by log binomial models. Significant results were based on 95% confidence intervals. Interaction terms were fit for household income by insurance to assess whether the relationship between visiting and insurance was modified by income.

The research was approved by the Human Research Ethics Committee of the University of Adelaide and participants gave informed consent prior to participation in the study.

## Results

### Response and sample characteristics

Responses were collected from n = 1,096 persons (response rate = 39.1%). Of these, 4% (n = 44) were edentulous or of unknown dentate status and were excluded from analyses. Thus, responses from n = 1,052 individuals were analysed in this study. The respondents showed a similar profile to comparable population survey data in terms of number of teeth, lower dentures, making a dental visit in the last 12 months, place of birth and education level (Table 1). However, a lower percentage of the study participants, compared to population estimates, were males, younger (aged 30–39 years), in the higher (>$100,000) income category, had dental insurance, or visited for a check-up at their last visit. Study participants also tended to have a higher percentage with upper dentures and more likely to speak English at home.

**Table 1:** Distribution of explanatory variables and comparison of dentate study participants with the population profile

	^**†**^**Census data**	^**‡**^**Population survey data**		***Study participants***	
Oral health status		%	(95% CI)	%	(95% CI)
Number of teeth – mean	-	25.2	(24.8, 25.6)	26.5	(26.1, 26.8)
Denture (upper jaw)	-	7.8	(6.8, 8.9)	11.7	(9.8, 13.8)
Denture (lower jaw)	-	2.1	(1.6, 2.7)	3.9	(2.8, 5.3)
Dental behaviour					
Last dental visit <12 months	-	60.5	(58.3,62.6)	59.7	(56.7, 62.7)
Check-up at last dental visit	-	57.2	(54.9, 59.5)	50.4	(47.4, 53.5)
Dental insurance	-	60.0	(57.9, 62.1)	53.9	(51.0, 57.0)
Socio-demographics					
Male sex	49.2	49.8	(47.6, 51.9)	42.3	(39.3, 45.4)
Age 30–39 years	34.4	34.1	(31.9, 36.4)	24.7	(22.1, 27.4)
Age 40–49 years	35.0	33.0	(31.1, 35.0)	32.9	(30.0, 35.8)
Age 50–61 years	30.6	32.9	(31.1, 34.8)	42.5	(39.4, 45.5)
Australian born	-	79.6	(77.8, 81.3)	80.7	(78.2, 83.1)
English main language at home	-	88.2	(86.6, 89.7)	94.6	(93.1, 95.9)
Education level of diploma or degree	-	44.6	(42.5, 46.8)	44.6	(41.5, 47.7)
Socio-economic status					
Household income < $60,000	-	24.2	(22.2, 26.2)	35.2	(32.2, 38.2)
Household income > $60,000–100,000	-	37.2	(35.1, 39.3)	32.2	(29.3, 35.1)
Household income > $100,000	-	38.7	(36.5, 40.9)	32.6	(29.7, 35.6)

†Census 2006: Australia, 30–59 year-olds [26].

‡National Dental Telephone Interview Survey 2010: Australia, dentate 30–61 year-olds.

### Effect of household income on the relationship between dental insurance and dental visiting

The study population distributions and dental visiting by sex, age, household income, tooth brushing and insurance status are presented in Table 2. Overall, 71.5% made regular dental visits and 50.4% last visited for a check-up. With the exception of one explanatory variable, sex, regular visits and visiting for a check-up were associated with all explanatory variables. Sex was associated with visiting for a check-up but was not associated with regular visiting (Table 2).

**Table 2:** Distributions and dental visiting by sex, age, income, tooth brushing, and insurance

	**Distributions**	**Regular dental visits**^**†**^			**Check-up visits**			**Adjusted effects: regular dental visits**			**Adjusted effects: check-up visits**		
	%	%	P value		%	P value		PR (95 % CI)	P value		PR (95 % CI)	P value	
**Sex**													
Male	42.3	69.2	0.1579	NS	46.8	0.0462	*	0.98 (0.91, 1.05)	0.5138	NS	0.89 (0.78, 1.01)	0.0688	NS
Female	57.7	73.2			53.1			Ref.			Ref.		
**Age group**													
30-39 yrs	24.7	67.6	0.0473	*	57.0	0.0486	*	Ref.			Ref.		
40-49 yrs	32.9	69.9			49.3			1.06 (0.96, 1.16)	0.2607	NS	0.85 (0.74, 0.98)	0.0214	*
50-61 yrs	42.5	75.7			47.6			1.11 (1.02, 1.21)	0.0154	*	0.83 (0.72, 0.95)	0.0084	**
**Household income**													
Up to $60,000	35.2	62.9	<0.0001	**	41.0	<0.0001	**	0.78 (0.65, 0.93)	0.0073	**	0.65 (0.50, 0.83)	0.0008	**
$60,001-100,00	32.2	73.9			52.0			0.85 (0.70, 1.04)	0.1154	NS	0.68 (0.51, 0.91)	0.0101	*
>$100,000	32.6	78.6			60.4			Ref.			Ref.		
**Tooth brushing**													
Twice a day or more	57.5	78.7	<0.0001	**	57.0	<0.0001	**	1.19 (1.10, 1.28)	<0.0001	**	1.26 (1.10, 1.44)	0.0006	**
Less than twice a day	42.5	62.2			41.7			Ref.			Ref.		
**Dental insurance**													
Insured	53.9	83.2	<0.0001	**	60.8	<0.0001	**	1.18 (1.01, 1.36)	0.0316	*	1.13 (0.92, 1.39)	0.2283	NS
Uninsured	46.1	57.7			38.3			Ref.			Ref.		
**Interaction**													
Up to $60,000 × Insured	-	-			-			1.28 (1.04, 1.56)	0.0177	*	1.33 (0.97, 1.82)	0.0754	NS
$60,001-100,00 × Insured	-	-			-			1.17 (0.95, 1.45)	0.1454	NS	1.40 (1.01, 1.94)	0.0425	*

Notes.

Chi-Square statistic, *(P < 0.05), **(P < 0.01).

PR: Prevalence Ratio.

†: On average visit a dental practitioner at least once every two years.

Overall, 53.9% of dentate adults were insured (Table 2). Proportion insured varied by household income with those in the lowest income group (33.5%) less likely to be insured than those in the $60,001 to $100,000 group (61.3%) and the highest income group (70.4%).

**Figure 1:**
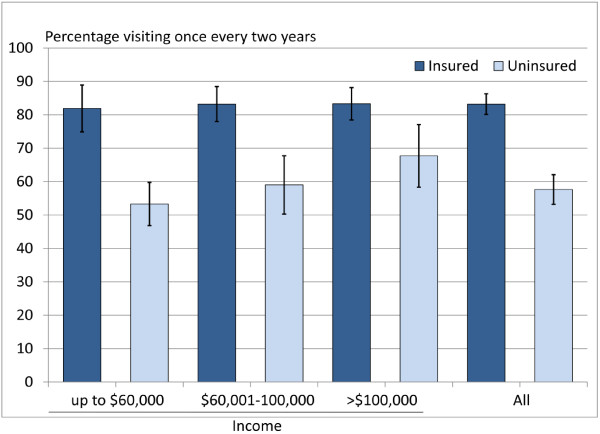
Regular dental visiting by insurance status and household income with 95% confidence intervals.

**Figure 2:**
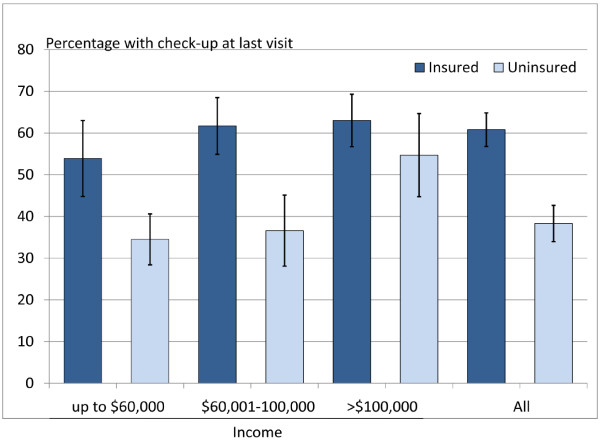
Visiting for a check-up by insurance status and household income with 95% confidence intervals.

The stratified bivariate associations are presented in Figures 1 and 2. The association between dental insurance and dental visiting varied across household income groups. Among those in the highest income group the proportion making regular visits and the proportion who visited for a check-up did not vary by insurance status. In contrast, for those in the lowest income group, the proportion making regular visits and visiting for a check-up was lower for uninsured adults than those who were insured, although differences in the proportion making regular visits was not significant (95% CIs overlapped) (Figure 1 and Figure 2).

After adjusting for explanatory variables the significant positive association between insurance status and regular dental visiting persisted. Insured individuals had a higher prevalence of visiting at least once every two years compared to participants without insurance (adjusted PR = 1.18; 95% CI: 1.01-1.36). Household income also remained positively associated with regular dental visiting, with participants in the lowest income group having a lower prevalence of regular dental visiting (adjusted PR = 0.78; 95% CI: 0.65-0.93) compared to those in the highest income group. Furthermore, the interaction between insurance and household income group, indicated in Figure 1, also persisted after controlling for other explanatory variables. The prevalence ratio of regular visiting was higher for insured adults in the lowest income group compared to insured in the highest income group (Table 2).

In terms of visiting a dentist for the purpose of a check-up, insurance status was not significantly associated (adjusted PR = 1.13; 95% CI: 0.92-1.39); however, household income was positively associated. Participants in the lowest income category had a significantly lower prevalence of last visiting for a check-up (adjusted PR = 0.65; 95% CI: 0.50-0.83) compared to those in the highest income group. Consistent with the pattern observed for regular visiting, there was a significant interaction between insurance status and household income, indicating that the associations between insurance and visiting for a check-up significantly vary by household income group (Table 2).

## Discussion

The key finding of this study was that household income modified the relationship between dental insurance and regular dental visiting and visiting for a check-up, with insurance status having a greater impact on visiting among lower income households.

The results of previous studies were consistent with the finding that household income is positively associated with regular visiting and visiting for a check-up [4,7,10,11,14]. Individuals from lower socioeconomic backgrounds were likely to lack the necessary economic resources to obtain regular preventive dental care [14]. Low income adults attending for care in public dental clinics often face long waiting periods, [18] and are more likely to receive emergency care rather than preventive care such as check-ups [16]. Similarly, adults from low income households were more likely to report that the cost of dental care prevented them from obtaining recommended treatment, such as regular check-ups [2,4]. Financial barriers also had an impact on the likelihood of regular dental visiting, with lower income individuals more likely to avoid or delay visiting a dentist until symptoms or problems arose [4,14,15].

The finding that dental insurance was positively associated with regular dental visiting was consistent with earlier findings [3-5,7-10]. This trend can be explained by the partial removal of financial barriers provided by dental insurance. Insured individuals were more likely to obtain recommended regular dental care than their uninsured counterparts, who may have avoided or delayed visiting a dentist until problems or symptoms appeared [4].

In this study household income was found to modify the association between insurance status and dental visiting. Specifically, dental insurance was associated with visiting behaviour to a greater extent among lower income groups. The higher percentage of regular dental visits and check-up visits observed for insured persons in the unadjusted analysis was less pronounced in the higher income group, but was more apparent in lower and middle income groups. Adjusted for other explanatory variables these associations remained significant. This suggests that dental insurance may be a more important enabling factor for access to dental care among lower income groups compared to higher income groups. This finding is consistent with a Canadian study which found that dental insurance was an important contributing factor to increased utilisation of dental services, and that this effect was most pronounced among lower income households [19,20]. Although adults from higher income households were found to be more likely to purchase dental insurance, [1,3,4,6,20] they derived fewer financial benefits from insurance compared to their lower income counterparts.

While over one-fifth of insured adults in Australia report financial barriers to receiving dental care, [21] which may in part be explained by the limited cost attenuation and relatively low annual claim limits provided by Australian dental insurance plans [9,22], nevertheless the persistent associations between having insurance and dental visiting after controlling for confounders supports the conclusion that dental insurance is an important enabling factor in dental visiting. These findings indicate that increasing coverage of dental insurance may improve access to care for lower income groups, but may have less influence on visiting patterns of higher income groups.

### Limitations

A limitation of this study was that household income does not take into account the number of people dependent on that income. However, clear gradients in dental visiting were observed across household income groups. While the response yield provided sufficient numbers for analysis, the response rate was low, particularly with multiple follow-ups [23]. The electoral roll should provide an adequate sampling frame for a population survey of adults. While a response rate of 60% may be considered adequate as a benchmark, [24] response rates require evidence to examine bias [25]. Key demographic characteristics of sex and age from the 2006 Census showed the study participants were less likely to be male and from the younger adult age group [26]. Other comparable population sample data also showed the survey respondents were more likely to be from the lower income group, and have lower levels of dental insurance and check-ups at the last dental visit. The cross-sectional nature of the analysis limits the ability to comment on the observed associations in terms of causal relationships.

While prevalence ratios of visiting may appear relatively low (Table 2), seemingly marginal PRs in relation to dental visiting can have a substantial impact on aggregate demand for dental visits. For example, ignoring the role of other unmeasured confounders, an 18% higher probability of regular visiting by insured adults would indicate that if uninsured Australian adults were to become insured, the resulting increase in demand for dental visits would be in excess of 1.8 million dental visits per annum (based on a uninsured population of 11.5 million people and 1.5 visits per capita).

## Conclusions

Although the results of this study should be interpreted with caution due to the aforementioned limitations, the findings showed that household income modified the relationships between insurance and regular visiting and visiting for a check-up. Dental insurance was found to have a greater effect on visiting among lower income groups. This finding has implications for oral health policy, suggesting that lower socioeconomic groups may derive greater benefit from dental insurance than their higher income counterparts.

## Competing interests

The authors declare that they have no competing interests.

## Authors’ contributions

All authors were involved in the analysis and interpretation of data, revising the paper and giving final approval for publication of the manuscript. OA prepared the draft manuscript, DB was involved in design of the project, and DT in acquisition and preparation of data. All authors read and approved the final manuscript.

## Pre-publication history

The pre-publication history for this paper can be accessed here:

http://www.biomedcentral.com/1472-6963/13/432/prepub
